# Endovascular Repair of a Traumatic Popliteal Artery Injury

**DOI:** 10.7759/cureus.31100

**Published:** 2022-11-04

**Authors:** Anita Nguyen, Tiziano Tallarita, Jason Beckermann, Joseph Wildenberg, Thomas Carmody

**Affiliations:** 1 Cardiothoracic Surgery, Mayo Clinic, Rochester, USA; 2 Vascular Surgery, Mayo Clinic, Eau Claire, USA; 3 Surgery, Mayo Clinic, Eau Claire, USA; 4 Interventional Radiology, Mayo Clinic, Eau Claire, USA; 5 Cardiothoracic Surgery, Mayo Clinic, Eau Claire, USA

**Keywords:** polytrauma patient, intraoperative/postoperative anticoagulation, popliteal artery, interventional radiology stent placement, extremity vascular trauma

## Abstract

In patients who sustain a traumatic arterial injury to the lower extremities, timely intervention is key for limb salvage. Traditionally, patients with a popliteal injury have undergone an open surgical bypass but, in recent years, endovascular repair has become more frequent. We present the case of a 46-year-old male who sustained a right tibial/fibular fracture and an associated popliteal artery injury during a pedestrian versus car accident. At presentation, distal signals were not detectable on duplex ultrasonography, and computed tomography confirmed an occlusion of the P3 popliteal artery and proximal anterior tibial and tibioperoneal trunk, as well as a comminuted tibia and fibula fracture. He also had a subdural hematoma without midline shift. He was taken to the operating room emergently and, following external fixation of the tibial/fibular fracture, he underwent angiography of the right leg. There was no thrombus or extravasation but a static column of blood secondary to a flow-limiting intimal flap was present, and an endovascular repair of the popliteal artery with balloon angioplasty and Tack stents (Intact Vascular, Wayne, PA) was pursued. Heparin was not utilized due to the patient's intracranial hemorrhage. On hospital day four, he underwent internal fixation of the tibial/fibular fracture. The subarachnoid/subdural hematoma remained stable and a prophylactic dose of rivaroxaban and aspirin was started. The patient recovered well from these procedures and was discharged 16 days after the accident.

## Introduction

Popliteal arterial trauma is relatively rare but carries a high risk for limb loss (up to 16%) and mortality (approximately 8%) [1-4]. In patients who sustain traumatic arterial injury to the lower extremities, timely intervention with rapid revascularization is key for limb salvage. Traditionally, these patients have undergone open surgical repair. However, the endovascular approach has been increasingly utilized because it has several potential advantages, including rapid vascular control of bleeding and vessel recanalization, no need to harvest the saphenous vein, decreased operating time, and improved surgical morbidity [5].

The Tack Endovascular System (Philips Medical, Amsterdam, Netherlands) has been approved for the treatment of post-balloon angioplasty dissection during the treatment of atherosclerotic disease, with 12-month primary patency of 76.4%, and freedom from target lesion revascularization of 89.5% [6,7].

This case report describes, for the first time, the use of tacking stents for a traumatic popliteal injury.

## Case presentation

**Figure 1:**
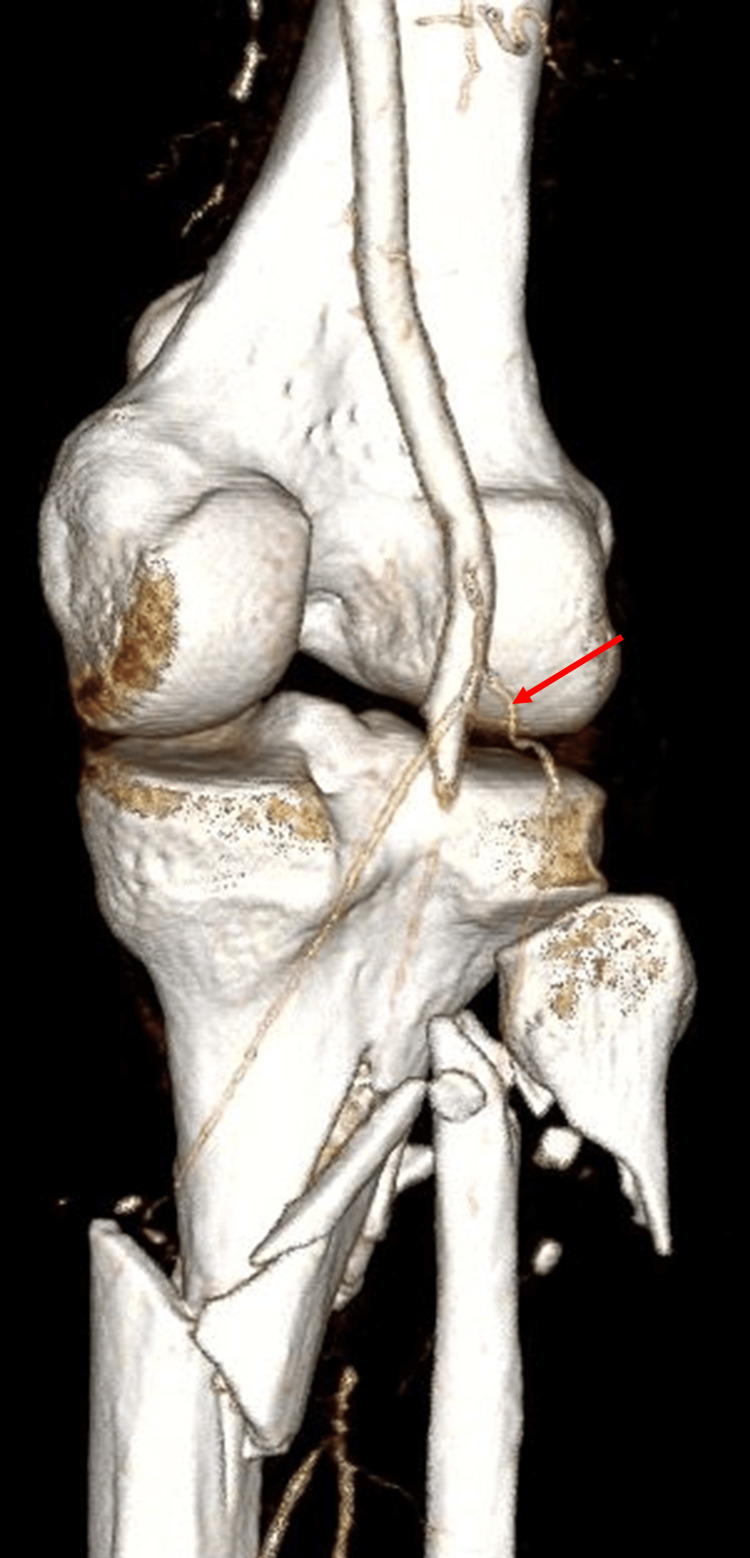
3D reconstructed computed tomography with arterial contrast showing the occlusion at the level of the right popliteal artery

A 46-year-old male presented to the emergency department after he was struck by a car traveling at 30-40 mph, hitting the windshield of the vehicle. On scene, the patient appeared confused with a Glasgow Coma Score of 14. He was transferred to the emergency department at our level II trauma center, where his initial vital signs were stable, with a blood pressure of 156/125 mmHg and a heart rate of 113. On examination, his right leg was deformed, with an apparent tibial/fibular fracture, pallor of the right foot, and no ultrasound signal in the posterior tibial (PT) or dorsalis pedis (DP) arteries. The patient complained of numbness and decreased motor function of the foot. After the initial resuscitation, computed tomography (CT) scans were performed, which revealed a 15-mm, right-sided subdural hematoma without significant mass effect, a right anterior sixth rib fracture, a right pubic ramus fracture, and a comminuted fracture of the right proximal tibia and fibula with loss of flow to the popliteal artery (Figure 1).

**Figure 2:**
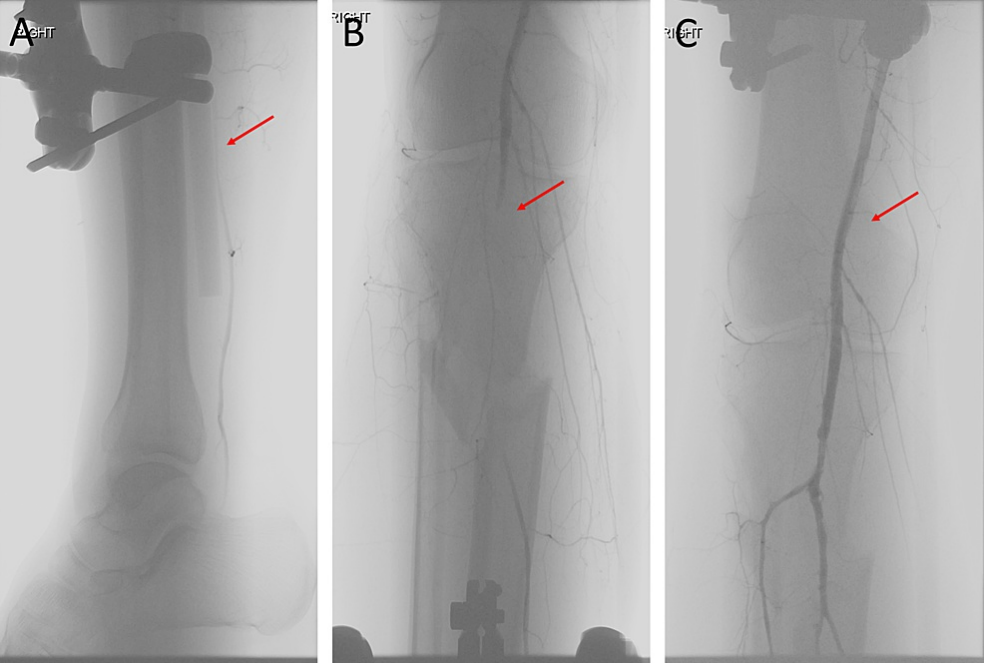
Initial angiography showing occlusion of the right popliteal artery, with some reconstitution of distal flow (A and B). Completion angiography showing Tacking stents placed in the popliteal artery and patent outflow (C)

These images were suggestive of dissection or transection of the distal popliteal and proximal tibial arteries. Orthopedic and vascular surgery were consulted and recommended external fixation of the tibial/fibular fracture with fasciotomies, followed by an on-table angiography, as well as consideration of a popliteal-tibial bypass using a contralateral great saphenous vein graft. Intraoperatively, a uniplanar external fixator was placed spanning the knee and pinned to the tibia and femur. Following external fixation, signals below the level of the knee continued to be non-detectable on duplex ultrasonography. An angiogram was performed via antegrade right common femoral access, showing abrupt interruption of flow at the level of the P3 popliteal artery segment (Figure 2).

**Figure 3:**
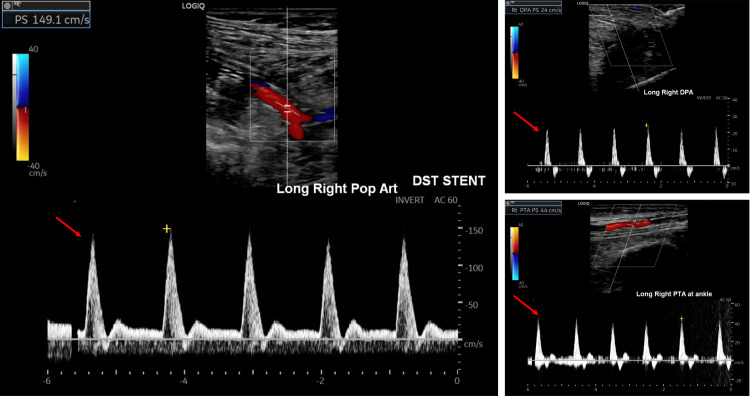
Duplex ultrasonography of the distal popliteal artery (A), distal posterior tibial artery (B), and dorsalis pedis artery (C) showing preserved triphasic waveforms throughout

The anterior and posterior tibial arteries were reconstituted in a retrograde fashion from collaterals coming from the genicular and profunda femoral arteries. Based on these findings, an attempt was made to repair the injury endovascularly. The injured popliteal segment was crossed intraluminally with a soft Glidewire and ballooned with a 4 mm balloon. A post-ballooning angiogram showed a localized dissection flap in the P3 popliteal segment without intraluminal thrombus. The decision was made to use the Tack endovascular system off-label to stabilize the flap. Three Tacking stents were deployed along different points of the dissection flap. The completion angiogram showed patent popliteal, anterior, peroneal, and posterior tibial arteries and an open pedal loop (Figure 2C). Prophylactic fasciotomies were performed to release all four compartments. The patient had palpable DP and PT pulses at the conclusion of the procedure. He returned to the operating room three days later to undergo an open reduction internal fixation of the tibia and fibula. Pulses remained palpable following internal fixation of the right leg. Based on a recent study in patients with peripheral vascular disease [8], low-dose aspirin (81 mg) and rivaroxaban (2.5 mg twice a day) were started on postoperative day 5, as the subdural/subarachnoid hematoma had remained stable. He was able to ambulate with physical therapy throughout his hospital stay and was discharged in stable condition 16 days after the accident. At the three-month follow-up, he was feeling well, and his DP and PT remained palpable. Arterial ultrasound showed a widely patent stent within the popliteal artery with no significant stenosis and triphasic distal waveforms (Figure 3).

Right ankle brachial indices were 1.14 (PT) and 1.09 (DP).

## Discussion

We present a novel use of Tacking stents to treat an isolated popliteal artery injury. This approach was feasible because the flow-limiting dissection was not associated with intraluminal thrombus. It provided rapid re-establishment of direct blood flow to the foot (Figure 2C) without the need to perform a bypass, avoiding incisional morbidity in a multiply injured trauma patient, and allowing repair without the need for systemic heparin in the setting of intracranial hemorrhage.

In general, the use of stents is avoided in arterial segments across the joints because of the numerous, multi-directional forces involved. The popliteal artery is subjected to frequent flexion and extension, raising significant concern for stent occlusion and/or fracture. In this case report, the use of such a short stent (1 cm long) as the Tacking stent potentially circumvents this problem. However, Tacking stents are used to purely control focal dissection flaps; in fact, they do not provide enough radial force to overcome stenosis and cannot be used in the presence of intraluminal thrombus. Specifically, the Tack endovascular system has been approved for the treatment of post-balloon angioplasty dissection during the treatment of atherosclerotic disease, with 12-month primary patency of 76.4%, and freedom from target lesion revascularization of 89.5% [6].

To the authors’ knowledge, this is the first time Tacking stents were used to treat a traumatic popliteal injury, circumventing the need to perform a bypass. Because of the lack of data on mid and long-term patency, the authors recommend frequent follow-ups with ultrasonography (every three months for the first year). If there is a concern for stenosis of the treated segment, a bypass with the saphenous vein is recommended in standard-risk patients. Further, endovascular repair may not be successful in patients with traumatic injuries due to complete popliteal transection.

In selected patients, the use of stents in the popliteal artery for aneurysm repair [9] or atherosclerotic disease [10] has shown similar patency to surgical bypass. Outside selected scenarios, the primary patency of a below-the-knee bypass with the saphenous vein remains superior [11,12]. A retrospective study of the National Trauma Data Bank of patients who suffered a blunt or penetrating injury of the superficial femoral artery and/or the popliteal artery reported an overall incidence of superficial femoral and/or popliteal arterial injury in 0.2% of all trauma patients [1]. Endovascular repair was performed in 5.7%, but interestingly, the authors noticed an increase in endovascular stent placement over the seven-year study period (3.2% in 2007 versus 7.6% in 2014), suggesting that endovascular repair is becoming more prevalent in the United States and Canada. However, endovascular approaches were performed more frequently in patients with superficial femoral artery injuries, and surgeons were more cautious to repair popliteal arteries endovascularly due to the higher association with limb loss.

Comparative studies of patients undergoing open versus endovascular repair are limited to small retrospective cohorts but risks of limb loss appear similar in patients undergoing endovascular versus open repair. Potter et al. reported that in-hospital amputation-free survival was similar between patients who underwent open versus endovascular repair, but their cohorts included patients with femoral and popliteal injuries [1]. Surprisingly, overall mortality was higher in those undergoing endovascular stenting while the rate of fasciotomy was lower. Ganapathy et al. reviewed a series of 68 patients who sustained peripheral arterial trauma to the upper or lower extremities and underwent endovascular versus open repair [13]. Consistent with the prior results, the patients who underwent endovascular repair were older but less likely to require fasciotomy. However, the authors concluded that lower risks for fasciotomy in the endovascular group were likely skewed by patients with upper extremity injuries who rarely require fasciotomy.

In patients with lower extremity vascular trauma, evidence that endovascular options result in lower risks of fasciotomy is less clear. Although this may be a benefit of the endovascular approach, patients who are at risk of compartment syndrome due to prolonged ischemia should undergo prophylactic fasciotomies, as was the case in our patient.

Long-term data on patients with popliteal artery trauma undergoing endovascular repair are limited. Jiang et al. reported on the four-year outcomes in a cohort of 46 patients following endovascular repair of traumatic popliteal injuries [14]. All of these patients underwent endovascular repair with 50-150 mm stents through an antegrade or combined approach. There were no deaths within 30 days of the operation and the limb salvage rate was 89.1%, with five patients requiring below-the-knee amputation due to irreversible ischemia. Stent patency rates were 75.3% at 12 months, 61.9% at 24 months, and 55.7% at 48 months. Similarly, Zhong et al. reported in-stent thrombosis in one out of seven patients who underwent endovascular stenting to the popliteal artery at 18 months following the initial injury [15]. Although in-stent thrombosis appeared higher in these patients, most of these complications were amenable to endovascular recannulation.

## Conclusions

This case report represents the first description of endovascular treatment of traumatic popliteal injury with Tacking stents. Strict follow-up is recommended because of the lack of mid and long-term patency. In selected patients, endovascular treatment of traumatic popliteal injury with stents is a feasible option, and short-term outcomes in small cohorts appear to show equivalent rates of limb loss compared with open surgery. As endovascular repair is becoming more utilized in the traumatic setting, long-term comparative studies are needed to further delineate which patients will benefit from endovascular versus open repair.
